# Referral patterns to outpatient child and adolescent mental health services and factors associated with referrals being rejected. A cross-sectional observational study

**DOI:** 10.1186/s12913-021-07114-8

**Published:** 2021-10-08

**Authors:** Anna Sofie Hansen, Cecilie Haugaard Christoffersen, Gry Kjaersdam Telléus, Marlene Briciet Lauritsen

**Affiliations:** 1 Aalborg University Hospital, Psychiatry, Mølleparkvej 10, 9000 Aalborg, Denmark; 2 Department of Clinical Medicine, Aalborg University, Sdr. Skovvej 15, 9000 Aalborg, Denmark; 3 Psychology, Department of Communication and Psychology, Aalborg University, Rendsburggade 14, 9000 Aalborg, Denmark

**Keywords:** Referral pattern, Referral decision, Rejection, Service use, Child psychiatry, Adolescent psychiatry

## Abstract

**Background:**

Outpatient child and adolescent mental health services (CAMHS) are faced with the challenge of balancing increasing demands with limited resources. An additional challenge is high rejection rates of referrals which causes frustration for referring agents and families. In order to effectively plan and allocate available resources within CAMHS there is a need for up-to-date knowledge on referral patterns and factors associated with rejection of referrals.

**Methods:**

In this cross-sectional observational study we did a retrospective review of all referrals (*n* = 1825) for children (0–18) referred for assessment at the outpatient CAMHS of the North Denmark Region in 2018.

**Results:**

The most common referral reasons to CAMHS were attention deficit disorder (ADHD/ADD) (27.9%), autism spectrum disorder (22.4%), affective disorders (14.0%) and anxiety disorders (11.6%). The majority of referrals came from general practitioners, but for neurodevelopmental disorders educational psychologists were the primary referral source. Re-referrals constituted more than a third of all referrals (35.9%). Children in care were overrepresented in this clinical sample and had an increased risk (Adj. OR 2.54) of having their referrals rejected by CAMHS. Referrals from general practitioners were also associated with an increased risk of rejection (Adj. OR 3.29).

**Conclusions:**

A high proportion of children with mental disorders have a repeated need for assessment by CAMHS. There is a need for future research on predictors of re-referral to outpatient services to identify potential targets for reducing re-referral rates as well as research on how to optimize service provision for children with a repeated need for assessment. General practitioners are the main gatekeepers to CAMHS and research on interventions to improve the referral process should be aimed towards general practitioners.

## Introduction

Childhood mental disorders are common with a worldwide prevalence of 13.4% [1] and more than 50% of life-time mental disorders have their onset before the age of 18 [2, 3] . Childhood mental disorders largely influence children and young people’s (CYP) health, education and well-being [4, 5] and have a high economic impact on society [6, 7]. In the past decades there has been a steep increase in referral rates to outpatient child and adolescent mental health services (CAMHS) [4, 8, 9] but research continues to document large unmet needs for CAMHS among CYP with mental disorders [9–11]. This leaves CAMHS in the dilemma of balancing existing resources with increasing demands as investments in CAMHS do not match the resources needed to provide services to all CYP in need [12, 13]. In order to effectively plan and allocate available resources within CAMHS we need current knowledge on referral patterns, but international research within this field is scarce [14].

Child mental health services are often organized in a stepped care approach [15] with multi-agency collaborations across social services, education and healthcare. The aim is to ensure that milder mental health problems are treated in primary care settings (general practitioners, education and social services) and only children with moderate to severe mental disorders are be referred to CAMHS [16, 17].

**Fig. 1:**
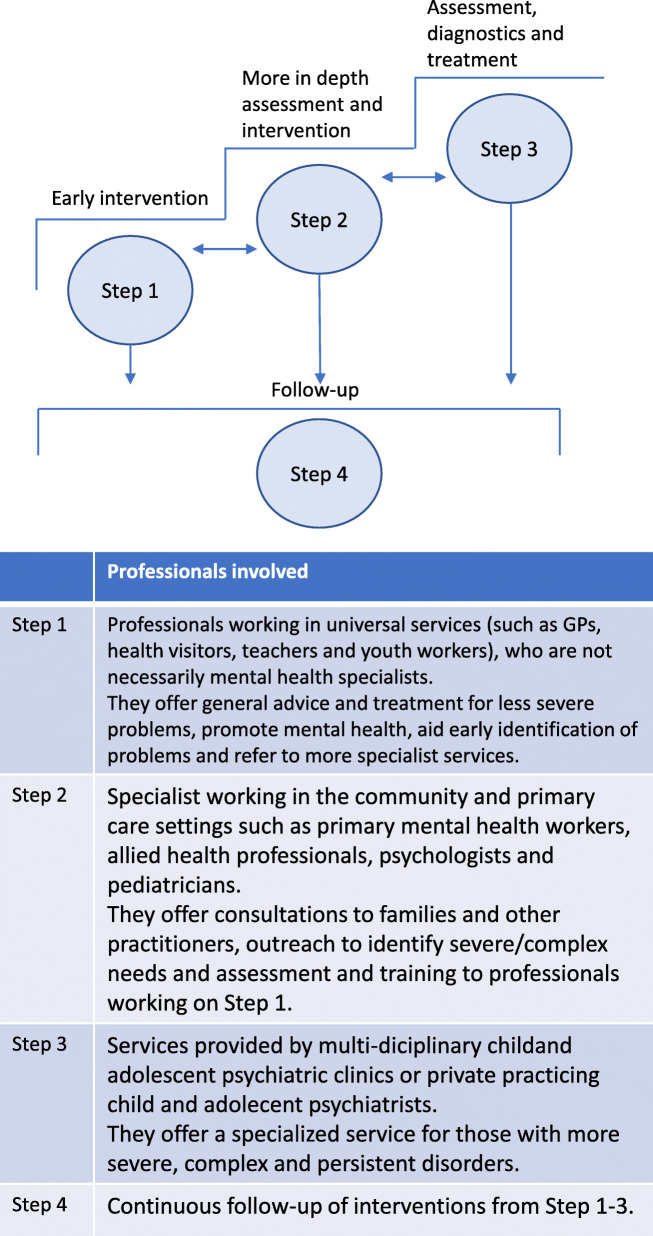
Danish model of stepped care for children with mental disorders. Model for stepped-care based on the model in the Danish Health Authorities disease management program for mental disorders in children and adolescents [17]

The Danish model of stepped care for CYP with mental disorders is outlined in Fig. 1 and is very similar to the tier model in the UK [18]. General practitioners (GPs) are the main gatekeepers to CAMHS [14, 18–21] in most European countries, but educational services also play an important role in the referral process [18, 21–23]. This potentially makes the referral process more complex. Professionals from primary settings, like GPs, and parents have expressed frustration with high rejection rates by CAMHS [24, 25] and studies have documented that GPs are uncertain about referral criteria to CAMHS [26]. In Denmark the rejection rate for referrals by CAMHS has been stable at around 20–25% for the last decade [27], which is in line with findings from other Scandinavian countries and the UK [20, 27, 28]. Despite the important role of the referral process in care pathways and the identified problems associated with referrals to outpatient services there has been very little research on interventions to improve the referral process [29]. In order to tailor interventions aimed at minimizing rejection rates, we first need to identify factors associated with increased risk of rejection. Very few studies have done so for CAMHS. Studies from the UK found increased odds of rejection for referrals from GPs [20] and teachers [18] as well as for referrals form emotional and behavioral difficulties [18]. Further research on who is rejected and why is still needed.

The aim of this study is to contribute knowledge on current referral pattern to outpatient CAMHS and investigate what characteristics are associated with rejection of referrals.

## Methods and materials

The study was a cross-sectional observational study. A retrospective systematic review was conducted of referral letters to outpatient CAMHS. The study was an exploratory study, with no hypothesis formulated in advance.

All referral letters for assessment of CYP under 18 years received at the CAMHS of the North Denmark Region from January–December 2018 were included in the study. Referrals with the purpose of treatment of an already diagnosed mental disorder were excluded from the study, as were referrals where it was not possible to determine if the purpose of the referral was assessment or treatment.

The study was conducted at the only CAMHS in the North Denmark Region. The CAMHS provides multidisciplinary specialist mental health services. The catchment area for the center covers both urban and rural areas with approximately 114,000 inhabitants between 0 and 18 years of age [30]. At the study center 96% of accepted referrals were seen for a first appointment within 30 days [31].

### Procedure

Referral information was extracted from the referral letters using a bespoke form developed for this study. The referral letters were reviewed by three graduate level psychology students after training by the first author (ASH), who is a 5th year child and adolescent psychiatric trainee. The graduate students could also consult with ASH when reviewing the referral letters.

### Variables

Information on *referral decision*, *placement in care* and any previous contacts to CAMHS in the North Denmark Region was obtained from the electronic records. In addition, referral letters were checked for information about *previous assessments* for a mental disorder conducted outside the study center. Sociodemographic characteristics included age, sex and placement in care.

*Referral source* was categorized into four categories: GPs, other medical doctor (private practicing specialist or hospital-based medical doctor), educational psychologist or case worker from social services.

*Primary referral diagnosis* was assigned according to ICD-10 [32] by a specialist in child and adolescent psychiatry to all referrals that were accepted to the CAMHS. If the referral was rejected, the referral diagnosis stated on the referral letter by the referring agent was taken as the primary referral diagnosis. In case of multiple referral diagnoses on a rejected referral, the primary referral diagnosis was decided by the first author (ASH) based on the referral letter. Primary referral diagnoses were divided into broader diagnostic categories as seen in Table 1. Specified referral diagnosis, that constituted less than 2.5% of the referrals were grouped together as “other”. These included psychoses, tic disorders, attachment disorder, conduct disorder and personality disorder.

**Table 1:** Characteristics of the sample

	Total sample		Accepted		Rejected		*P*-value
	N	%	N	%	N	%	
	1825	100	1363	74.7	462	25.3	
**Sociodemographic characteristics**							
Boys	989	(54.2)	745	(54.7)	244	(52.8)	0.49
Median age (IQR)	13.5	(9.5–16.0)	13.6	(9.6–16.0)	13.3	(8.8–15.7)	0.07
Placement in care	135	(7.4)	79	(5.8)	56	(12.1)	**< 0.001**
**Previous contact for mental health problems**							
Previously referred, but referral rejected	202	(11.1)	144	(10.6)	58	(12.6)	0.24
Previously assessed for a mental disorder	452	(24.8)	357	(26.2)	95	(20.6)	**0.02**
**Referral source**							
General practitioner	983	(53.9)	638	(46.8)	345	(74.7)	**< 0.001**
Other medical doctor	173	(9.5)	141	(10.3)	32	(6.9)	**0.04**
Educational psychologist	568	(31.1)	506	(37.1)	62	(13.4)	**< 0.001**
Case worker social services	101	(5.5)	78	(5.7)	23	(5.0)	0.56
**Primary referral diagnosis**							
Affective disorder	255	(14.0)	213	(15.6)	42	(9.1)	**< 0.001**
Anxiety disorder	212	(11.6)	149	(10.9)	63	(13.6)	0.12
Reactions to severe stress and adjustment disorders	98	(5.4)	89	(6.5)	9	(2.0)	**< 0.001**
Eating disorder	90	(4.9)	70	(5.1)	20	(4.3)	0.49
Autism spectrum disorder	409	(22.4)	351	(25.8)	58	(12.6)	**< 0.001**
Attention Deficit disorder (ADHD/ADD)	509	(27.9)	385	(28.3)	124	(26.8)	0.56
Other	168	(9.2)	101	(7.4)	67	(14.5)	**< 0.001**
Unspecified	84	(4.6)	5	(0.4)	79	(17.1)	**< 0.001**

*Previous support/interventions* were documented based on information from the referral letters. Previous support/interventions were subdivided into support/interventions provided in school, psychosocial support/interventions provided outside of school and support/interventions provided by healthcare professionals. Allied health professionals included physiotherapists, occupational therapists, speech therapists, health visitors and dieticians.

Descriptions of *cognitive level of functioning* were also extracted from the referral letter, as was *impact of mental health problems on schooling* and information about *self-harm or suicidal ideations*.

*Impact on schooling* included academic problems, absence from school on some days and complete school absence for the CYP and was included as a proxy variable for functional impairment caused by the CYP’s mental health problems.

### Statistical analysis

Descriptive statistics are reported as N (%) for all categorical variables and as median (interquartile range (IQR)) for referral age. For categorical variables, the Chi-squared test was applied. ANOVA was used for continuous variables. Post-hoc pairwise comparisons were made to investigate for differences in referrals for the four most common primary referral diagnoses.

Logistic regression was used to examine factors associated with a referral being rejected. To check for reliability of the data extraction 20 randomly selected referral letters reviewed by each of the graduate students were also reviewed by ASH. Reliability of the data extraction from the referral letters was calculated using an average of Cohen’s Kappa for all extracted variables comparing each rater to ASH.

The level of statistical significance was set at 5% for all analyses. All statistical analyses were executed using Stata Statistical Software: Release 15. College Station, TX: StataCorp LLC.

## Results

Overall reliability for the data extraction was moderate to strong with an average of kappa values for all computed variables between 0.77–0.81.

### Sample

**Fig. 2:**
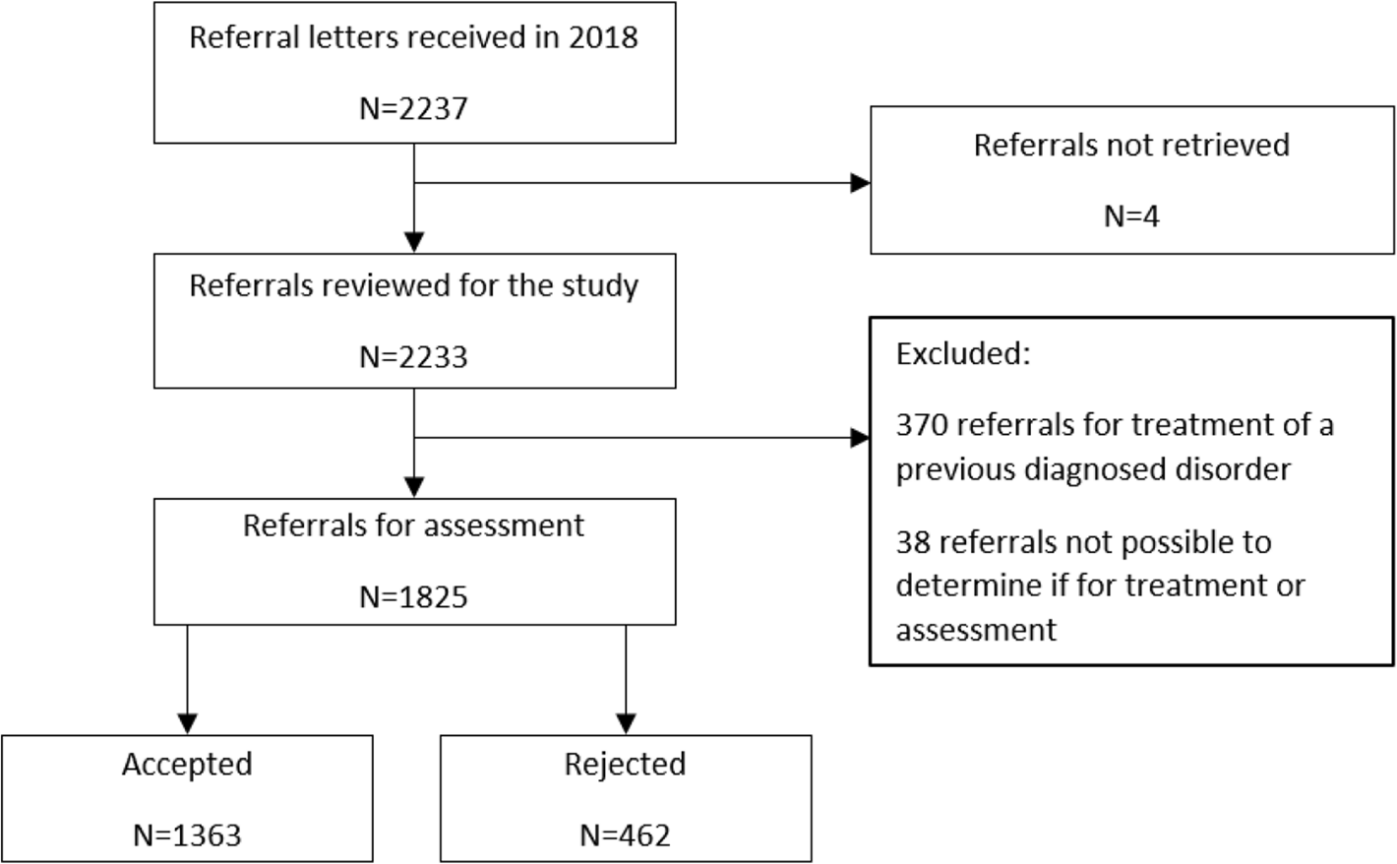
Study sample

In 2018, CAMHS in the North Region of Denmark received 2237 referral letters to outpatient services (Fig. 2). Of these four referral letters could not be retrieved, 370 were referred for treatment of a previously diagnosed mental disorder, and for 38 referral letters it was not possible to determine if the referral was for assessment or treatment of an existing disorder. Thus, 1825 referrals were included in the study. Of these 74.7% (*n* = 1363) were accepted for assessment and 25.3% (*n* = 462) were rejected.

### Referral pattern

As seen in Table 1, just over half (54.2%) of the referrals were for boys. The median referral age was 13.5 years (IQR 9.5–16.0). Referred girls were significantly older (median 14.8, IQR 12.2–16.5) than the referred boys (median age 11.5, IQR 8.1–15.0) (*p* < 0.001). CYP placed in care constituted 7.4% of the sample. More than a third of the referrals were re-referrals (35.9%), and 24.8% had previously been assessed for a mental disorder. The majority of the referrals came from GPs (53.9%), followed by educational psychologists (31.1%). The four primary referral diagnoses were attention deficit disorder (ADHD/ADD, 27.9%), followed by autism spectrum disorder (ASD, 22.4%), affective disorder (14.0%), and anxiety disorder (11.6%).

### Differences in referrals for the four most common primary referral diagnoses

As seen in Table 2 there were significant sociodemographic differences between the referrals for the four most common primary referral diagnoses. CYP referred for ADHD/ADD were statistically significantly more often placed in care compared to CYP referred for ASD or anxiety disorders (*p* = 0.02) (Table 2). Medical doctors (GP or other) referred 95.3% of all referrals for affective disorders and 88.3% of referrals for anxiety disorders, whereas educational psychologists were the primary referral source for referrals for ASD (58.4%) and ADHD/ADD (47.7%).

**Table 2:** Content of referral letters for the four most common primary referral diagnoses

	Affective disorder		Anxiety disorder		ASD		ADHD/ADD	
	N	%	N	%	N	%	N	%
	255	(14.0)	212	(11.6)	409	(22.4)	509	(27.9%)
**Sociodemographic characteristics**								
Boys	80	(31.4)	84	(39.6)	311	(76.0)	338	(66.4)
Referral age, median (IQR)	16.1	(14.5–17.1)	14.8	(12.1–16.4)	10.7	(7.2–14.1)	10.6	(8.0–14.8)
Placement in care	16	(6.3)	6	(2.8)	15	(3.7)	38	(7.5)
**Referral source**								
- General Practitioner	288	(89.4)	164	(77.4)	93	(22.7)	177	(34.8)
- Other medical doctor	15	(5.9)	23	(10.8)	48	(11.7)		(10.6)
- Educational psychologist	7	(2.8)	20	(9.4)	239	(58.4)	54	(47.7)
- Case worker social services	5	(2.0)	5	(2.4)	29	(7.1)	243 35	(6.9)
**Previous support/interventions**								
Combined (school, psychosocial and healthcare)	173	(67.8)	159	(75.0)	385	(94.1)	457	(89.8)
In school								
- Extra attention from teacher	16	(6.3)	17	(8.0)	22	(5.4)	35	(6.9)
- Educational psychologist involved	37	(14.5)	41	(19.3)	193	(47.2)	206	(40.5)
- Part time support teacher in class	≤4	(≤1.6)	9	(4.3)	48	(11.7)	62	(12.2)
- Full time special needs education	18	(7.1)	26	(12.3)	93	(22.7)	112	(22.0)
Psychosocial								
- For the child	105	(41.2)	89	(42.0)	76	(18.6)	86	(16.9)
- For the parents	14	(5.5)	10	(4.7)	48	(11.7)	82	(16.1)
- For both the child and parents	5	(2.0)	6	(2.8)	9	(2.2)	18	(3.5)
Healthcare								
- Allied health professionals	4	(1.6)	7	(3.3)	97	(23.7)	73	(14.3)
- Medical doctor	44	(17.3)	55	(25.9)	108	(26.4)	135	(26.5)
**Description of cognitive level**								
- Academic level	37	(14.5)	39	(18.4)	31	(7.6)	37	(7.3)
- Cognitive testing	11	(4.3)	32	(15.1)	281	(68.7)	321	(63.1)
**Impact on schooling**								
- Academic problems	12	(4.7)	6	(2.8)	83	(20.3)	138	(27.1)
- Absence on some days	46	(18.0)	37	(17.5)	48	(11.7)	68	(13.4)
- Complete school absence	34	(13.3)	45	(21.2)	41	(10.0)	19	(3.7)
**Self-harm and suicidal ideations**								
- Self-harm	20	(7.8)	7	(3.3)	21	(5.1)	25	(4.9)
- Suicidal ideations	105	(41.2)	18	(8.5)	29	(7.1)	26	(5.1)
- Self-harm and suicidal ideations	51	(20.0)	4	(1.9)	9	(2.2)	14	(2.8)

*ASD* Autism Spectrum Disorders, *ADHD/ADD* Attention Deficit Hyperactivity Disorder/Attention Deficit Disorder

When looking at previous support/interventions described in the referral letter, there were also significant differences (Table 2). Almost all CYP referred for assessment of ASD (94.1%) and ADHD/ADD (89.8%) had received some form of support/intervention prior to referral, which was statistically significantly (*p* < 0.001) more frequently than for CYP referred for anxiety disorders (75.0%) and affective disorders (67.8%). The type of support/intervention received prior to referral also differed according to primary referral diagnosis. Descriptions of support/interventions in school were present in less than 30.0% of referrals for affective disorders, whereas school interventions were mentioned in 87.0% of referrals for ASD. More than a fifth of the children referred for assessment for ASD or ADHD/ADD were in full time special needs education programs at the time of referral, compared to only one in 14 of children and adolescents referred for affective disorders. For referrals for both affective disorders and anxiety disorders, psychosocial interventions were mentioned in almost half of the referral letters, with the intervention most commonly aimed at the child (41.2% for affective disorders and 42.0% for anxiety disorders). Description of previous psychosocial interventions were statistically less common in referrals for ASD and ADHD/ADD (*p* < 0.001) compared to referrals for affective disorders and anxiety disorders, while psychosocial interventions aimed at the parents were statistically significantly more common in referrals for ASD and ADHD/ADD (*p* < 0.001). Descriptions of previous interventions by allied health professionals were most common in referrals for autism (23.7%). In 21.2% of referrals for anxiety disorders the referral letter mentioned that the CYP was not currently in school which was only the case in 10.0% of referrals for ASD and 3.7% for ADHD.

### Factors associated with rejection of referral from CAMHS

Table 3 shows the association between different factors and the decision by CAMHS to reject a referral. The adjusted logistic regression model was adjusted for age, sex, placement in care, previous contacts for mental health problems, referral source, primary referral diagnosis, previous support/interventions (in school, psychosocial, healthcare), description of cognitive level, impact on schooling and self-harm/suicidal ideations. We found no association between age or sex and rejection of referral (data not shown).

**Table 3:** Logistic regression model: factors associated with rejection of referral from CAMHS

	Rejected (unadjusted)		Rejected (adjusted)^a^	
	OR	95%CI	OR	95%CI
**Placement in care**				
- Lives with parents	Reference		Reference	
- Placed in foster care or residential home	**2.24**	**(1.56–3.21)**	**2.54**	**(1.61–4.00)**
**Previous contact for mental health problems**				
- First time referral	Reference		Reference	
- Previously rejected	1.12	(0.81–1.57)	1.23	(0.85–1.78)
- Previously assessed	**0.74**	**(0.57–0.96)**	**0.71**	**(0.52–0.98)**
**Referral source**				
- General practitioner	**3.35**	**(2.65–4.24)**	**3.29**	**(2.35–4.61)**
- Other medical doctor	**0.67**	**(0.45–0.99)**	0.64	(0.40–1.02)
- Educational psychologist	**0.26**	**(0.20–0.35)**	**0.30**	**(0.21–0.45)**
- Social worker	0.86	(0.54–1.39)	0.81	(0.48–1.38)
**Primary referral diagnosis**				
- Affective disorder	**0.54**	**(0.38–0.77)**	**0.37**	**(0.25–0.55)**
- Anxiety disorder	1.29	(0.94–1.76)	1.00	(0.70–1.45)
- Adjustment disorder	**0.28**	**(0.14–0.57)**	**0.23**	**(0.11–0.46)**
- Eating disorder	0.84	(0.50–1.39)	**0.52**	**(0.30–0.90)**
- Autism	**0.41**	**(0.31–0.56)**	**0.54**	**(0.38–0.76)**
- ADHD/ADD	0.93	(0.73–1.18)	1.18	(0.90–1.55)
- Other	**2.12**	**(1.52–2.94)**	**1.76**	**(1.22–2.54)**
**Previous support/interventions**				
In school				
- No support	Reference		Reference	
- Extra attention from teacher	0.66	(0.41–1.04)	1.18	(0.65–2.15)
- Educational psychologist involved	**0.53**	**(0.41–0.68)**	0.96	(0.68–1.36)
- Part time support teacher in class	**0.32**	**(0.20–0.54)**	**0.53**	**(0.30–0.95)**
- Full time special needs education program	**0.50**	**(0.36–0.68)**	0.86	(0.56–1.30)
Psychosocial				
- No interventions	Reference		Reference	
- for the child	**0.61**	**(0.47–0.80)**	0.83	(0.61–1.13)
- for the family/parents	**0.69**	**(0.48–0.99)**	0.98	(0.65–1.49)
- for both the child and the family/parents	0.79	(0.41–1.53)	1.56	(0.75–3.22)
Healthcare				
- No support	Reference		Reference	
- Allied health professionals	**0.48**	**(0.30–0.77)**	**0.60**	**(0.37–0.99)**
- Medical doctor	**0.67**	**(0.51–0.89)**	**0.41**	**(0.18–0.89)**
- Allied health professionals + medical doctor	**0.31**	**(0.15–0.67)**	**0.70**	**(0.51–0.95)**
**Description of cognitive level of functioning**				
None	Reference		Reference	
Cognitive testing	0.31	(0.24–0.40)	**0.34**	**(0.23–0.50)**
**Impact on schooling**				
No description	Reference		Reference	
Academic problems	**0.49**	**(0.35–0.69)**	0.81	(0.55–1.18)
Absence on some days	**0.72**	**(0.52–0.98)**	0.90	(0.63–1.27)
Complete school absence	**0.55**	**(0.37–0.82)**	0.85	(0.54–1.35)
**Self-harm and suicidal ideations**				
No description	Reference			
Self-harm without suicidal ideations	1.04	(0.66–1.62)	0.83	(0.50–1.40)
Suicidal ideations	**0.69**	**(0.49–0.98)**	0.80	(0.54–1.19)

^a^Adjusted for age, sex, placement in care, previous contacts for mental health problems, referral source, primary referral diagnosis, previous support/interventions, description of cognitive level, impact on schooling and self-harm/suicidal ideations

Referrals for CYP in care had a statistically significant increased risk of being rejected (Adj. OR 2.54, 95%CI 1.61–4.00). Referrals from GPs (Adj. OR 3.29, 95%CI 2.35–4.62) and referrals with “other” (Adj. OR 1.76, 95%CI 1.22–2.54) as the primary referral diagnosis also had an increased risk of being rejected. Previous assessment for a mental disorder was associated with decreased risk of rejection (Adj OR 0.71, 95%CI 0.52–0.98) as was being referred by an educational psychologist (Adj. OR 0.30, 95%CI 0.21–0.45). Several primary referral diagnoses were associated with decreased risk of rejection (Table 3).

Part time support teacher in the class (Adj. OR 0.53, 95%CI 0.30–0.95) and intervention by health professionals (Adj. OR 0.60, 95%CI 0.37–0.99 for allied health professionals and Adj. OR 0.41, 95%CI 0.18–0.89 for medical doctor) were the only previous support/interventions associated with decreased risk of rejection in the adjusted regression analyses. Description of previous cognitive testing was also associated with decreased risk of rejection (Adj. OR 0.34, 95%CI 0.23–0.50).

## Discussion

In this explorative cross-sectional study, we investigated referral patterns to outpatient CAMHS, and factors associated with referrals being rejected from CAMHS.

The sex and age distribution found in our sample is similar to that found in other European clinical samples [14, 18, 21]. In the following key findings from the study will be discussed.

### CYP placed in care

CYP placed in care were overrepresented in this study constituting 7.4% of referrals compared to only 1% in the background population in Denmark [33]. This is in line with previous findings by Larsen et al. who also reported high service use for this group, but also large unmet needs [34]. Another key finding was that CYP placed in care had a 2.54 increased risk of their referral being rejected compared to CYP living at home. Previous studies have documented that the prevalence for mental disorders in CYP placed in care is almost four-fold greater than in the general population [35, 36]. A systematic review of outcomes for CYP in care in the Nordic countries found that as young adults, CYP placed in care, had an increased risk of mental health problems, suicidal behavior and higher mortality in young adulthood compared to the general population [37]. Given this knowledge it is worrisome, that being placed in care is associated with increased risk of being rejected by CAMHS.

### Re-referrals

More than a third of referrals to CAMHS were re-referrals. This is comparable to previous findings from the UK [20] and Canada [38, 39]. Despite high re-referral rates, there is a paucity of research on predicters of re-referral to outpatient CAMHS [40]. There are several possible explanations for the high proportion of re-referrals. The nature of childhood mental disorders is that they are often persistent [10, 38] and recurrence rates for disorders such as depression and anxiety disorders are high [41]. In addition heterogeneity in psychopathological development [42] can result in a need for renewed assessment by CAMHS. Re-referrals as a result of the nature of childhood mental disorders are probably not preventable. However, there could be other preventable contributing factors. Co-morbidity rates for childhood mental disorders are high [43] and although comorbid conditions might not develop until after initial assessment, there is also the possibility that co-morbidity is missed at initial assessment. Structured diagnostic interviews have been shown to increase the likelihood of clinicians diagnosing comorbid disorders [44–46]. However, structured diagnostic interviews are often not used in routine clinical assessment [47–49]. This is relevant because overlooked disorders could have a negative effect on the treatment outcome for the child [49]. Development of co-morbid disorders post-assessment could be a result of insufficient service provision in primary settings following diagnosis of the primary disorder. Studies from in-patient setting have found an association between lower levels of aftercare [50] and delay in provision of aftercare services [51] and increased risk of re-admission. There is a need for more research on predictors of re-referral to outpatient CAMHS to identify targets for improvement of service provision for CYP with mental disorders. Regardless of the explanation, the high referral rates call for research on how to optimize services for children with a repeated need for referral to CAMHS [39]. With the knowledge of high comorbidity rates, it is positive that we found that previously having been assessed for a psychiatric diagnosis is associated with a decreased risk for a referral being rejected.

### Referral source

Referral from a GP was associated with a three times higher risk of the referral being rejected. This is relevant as GPs were responsible for the majority of referrals. The finding is in line with a study by Hinrichs et al., but contrasts with findings by Smith et al. who found increased risk of rejection for referrals from teachers compared to GPs [18, 20]. In Denmark teachers can not refer directly to CAMHS, so any referral from the educational system comes from educational psychologists. Teachers have limited training in child mental health [52], whereas educational psychologists have more in-depth knowledge of child mental health problems. Results from this study, indicates that referrals from professionals with more specialized knowledge are more often accepted by CAMHS than referrals by GPs. This might be due to these professionals having more time and options for both assessment and interventions prior to referral to CAMHS in accordance with the stepped care policy. However, involvement of professionals at a higher level in the stepped-care model prior to referral to CAMHS also requires a referral and may delay necessary assessment by CAMHS. GPs have expressed frustration with frequent rejections by CAMHS and a lack of clarity about the organization of mental health services for CYP [24]. In a systematic review of barriers to managing CYP mental health problems in primary health care settings O’Brien et al., identified lack of knowledge, skills, tools, time and resources as barriers for GPs referring to CAMHS [53]. In addition, they identified a desire from GPs for feedback on referrals as well as clearer referral criteria [53]. There is a need for further research to investigate why the rejection rate for referrals from GPs is so much higher than for other referral sources. Further knowledge on whether the higher rejection rates are due to inappropriate referrals of CYP to CAMHS or lack of information in the referral due to lack of skills and tools for correctly describing symptoms of mental disorders in CYP is needed. In case missing information is the primary problem, the use of web-based diagnostic interviews like the Development and Well-being Assessment (DAWBA) [54] might be a useful tool.

### Primary referral diagnosis

Referrals for affective disorder, reactions to severe stress and adjustment disorders and eating disorders were all associated with decreased risk of being rejected. It may be that these disorders all have a clearer onset of symptoms, with a distinctive change in the CYP’s emotional state and/or behavior making them easier to identify as psychiatric symptoms. In addition, there could be a more acute need for treatment, where it is not possible to await the potential effect of interventions in primary settings. It is less clear why referrals for ASD are associated with decreased risk of rejection, when ADHD is not. Possibly the symptomatology of ASD, in contrast with that of ADHD/ADD, is more clearly distinguishable from symptoms of conduct disorders and learning disabilities, which in Denmark are primarily treated within educational and social services. Another explanation might be that children with ASD are perceived to be more in need of an assessment by CAMHS to access necessary services from other sectors.

### Previous support/interventions

We found that the majority of CYP referred to CAMHS had received support/intervention prior to referral, which is in line with the stepped care model for child mental health services [16, 17]. However, a surprising finding from this study was that descriptions of most previous support/interventions in schools and all psychosocial support/interventions were not associated with a reduced risk of the referral being rejected. This finding might be due to a larger emphasis by CAMHS on psychopathology, i.e. if there are clear descriptions of psychiatric symptoms matching a moderate to severe mental disorder, than on whether interventions in primary settings have been attempted prior to referral. The same explanation might apply to why we also found no association between description in the referral letter of impact on schooling (i.e. academic problems or absence) or self-harm and the risk of rejection.

### Strengths and limitations

To our knowledge this is the largest study to date investigating factors associated with rejection of referrals to CAMHS. The systematic review of the content of the referral letters is a strength compared to previous studies of referral patterns.

However, there are also some limitations to the study that should be mentioned. The study was carried out at a single CAMHS and the results might not be representative of other CAMHS. The primary referral diagnoses are not verified referral diagnoses and other countries might have different ways of classifying referral reasons to CAMHS making it hard to make direct comparisons across studies. We were not able to check if previous interventions were omitted from the referral letter which means the results regarding previous support/interventions could be underestimating the proportion of referred CYP who did receive support prior to referral. However, the results from this study with regards to the percentages of referred CYP with different referral diagnoses in full time special educational needs programs are in accordance with previously published national reports from Denmark [55].

## Conclusion

GPs are the main gatekeepers to CAMHS, but educational services play an important role in referring children for neurodevelopmental disorders. Most CYP referred to CAMHS have received support/interventions from other services prior to referral in accordance with stepped care models. There is a need for further research to determine if the higher rejection rates for referrals from GPs are due to incorrect referrals for children not in need of CAMHS or due to missing information in the referrals. In either case, the findings indicate that there is a need to increase the knowledge of child mental disorders and child mental health services among GPs. Further research is also needed to shed light on why placement in care is associated with increased risk of rejection of referrals to CAMHS, as this is a vulnerable group with very high prevalence of mental disorders.

## Data Availability

Data is available upon reasonable request to the corresponding author.

## Abbreviations

CAMHS: Child and adolescent mental health service
ADHD/ADD: Attention deficit hyperactivity disorder/Attention deficit disorder
ASD: Autism spectrum disorder
CYP: Children and young people
GP: General practitioner
ICD-10: International classification of diseases version 10
IQR: Interquartile range
ANOVA: Analysis of variance
UK: United Kingdom
DAWBA: Development and Well-Being Assessment
